# Novel Amdovirus in Gray Foxes

**DOI:** 10.3201/eid1710.110233

**Published:** 2011-10

**Authors:** Linlin Li, Patricia A. Pesavento, Leslie Woods, Deana L. Clifford, Jennifer Luff, Chunlin Wang, Eric Delwart

**Affiliations:** Blood Systems Research Institute, San Francisco, California, USA (L. Li, E. Delwart);; University of California, San Francisco (L. Li, E. Delwart);; University of California, Davis, California, USA (P.A. Pesavento, D.L. Clifford, J. Luff);; California Animal Health and Food Safety Laboratory, Davis (L. Woods);; California Department of Fish and Game, Rancho Cordova, California, USA (D.L. Clifford);; Stanford Genome Technology Center, Stanford, California, USA (C. Wang)

**Keywords:** viruses, amdovirus, Aleutian mink disease virus, parvovirus, gray fox, dispatch

## Abstract

We used viral metagenomics to identify a novel parvovirus in tissues of a gray fox (*Urocyon cinereoargenteus*). Nearly full genome characterization and phylogenetic analyses showed this parvovirus (provisionally named gray fox amdovirus) to be distantly related to Aleutian mink disease virus, representing the second viral species in the *Amdovirus* genus.

Aleutian mink disease virus (AMDV) is currently the only member of the genus *Amdovirus* in the family *Parvoviridae*; it can infect diverse breeds of farmed and feral mink, in addition to other mustelids (e.g., ferrets, otters), raccoons, and foxes (*1**,**2*). AMDV has an ≈5-kb single-stranded DNA genome and, like other parvoviruses, replicates through a rolling-hairpin mechanism (*3*). The viral genome has 2 large open reading frames (ORFs), encoding nonstructural (NS1, NS2, putative NS3) and structural viral proteins (VP1 and VP2). Alternative splicing enables expression of multiple messenger RNAs (*4*). AMDV strains can exhibit sequence variability in their NS gene, and 3 genetic groups have been identified on the basis of partial nucleotide sequences of this region (*5*).

AMDV infection can cause an acute and fatal interstitial pneumonia in newborn mink. It can also cause a chronic disorder of the immune system in adult mink, characterized by persistent viral infection, plasmacytosis, hypergammaglobulinemia, and immune complex–mediated glomerulonephritis and arteritis, resulting in major economic losses to mink farms (*6*). AMDV infection can also be asymptomatic. The different outcomes are determined by host factors that include age, immune status, and the virulence of the virus strains (*7**,**8*). AMDV can be transmitted through urine, feces, and saliva as well as vertically in utero (*9**,**10*). In 1 report, a ferret found to be naturally infected with AMDV showed acute dyspnea and posterior paresis with histopathologic lesions similar to those seen in mink; the ferret became comatose and died (*11*). Recently, 2 mink farmers with vascular disease and microangiopathy, similar to conditions in mink with Aleutian disease, were found to have AMDV-specific antibodies and were AMDV DNA positive, suggesting a potential relationship between AMDV and human symptoms (*12*).

We used random PCR amplification and high-throughput sequencing technology to investigate viral sequences found in the spleen and lung tissues of a sick gray fox (*Urocyon cinereoargenteus*) from California. A highly divergent amdovirus was identified, and the near full genome of this virus was obtained. Phylogenetic analysis indicated that this virus, designated as gray fox amdovirus, is a new amdovirus species, only the second for that genus.

## The Study

The gray fox studied here was identified during the summer of 2009 in Sonoma County, California. It had severe gait abnormalities, lymphadenopathy, and acute muscle inflammation, and was euthanized at a wildlife rehabilitation center. Using the generic viral particle enrichment method previously described for tissues (*13*), we generated ≈14,000 sequence reads from spleen and lung samples. We found 136 sequence reads in spleen tissue that were related to AMDV by using BLASTx (E score <10^–5^) (www.ncbi.nlm.nih.gov/BLAST); these could be assembled into 24 contigs covering ≈60% of the viral genome. By connecting gaps between sequenced viral fragments and amplifying the genome extremities by using PCR primers based on AMDV sequences, the nearly complete genome of the new amdovirus (GenBank accession no. JN202450) was acquired. We temporarily named it gray fox amdovirus (GFADV).

**Figure 1:**
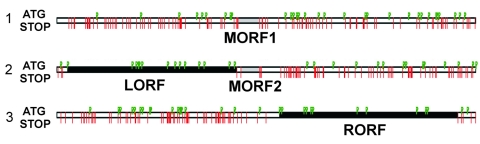
Open reading frames (ORFs) in gray fox amdovirus genome. Three possible reading frames of the plus-strand sequence with the stop codons indicated by red lines and ATG codons by green flags. Two major ORFs, left (LORF) and right (RORF), are indicated by black bars; 2 small middle ORFs (MORF1 and MORF2) are indicated by gray bars.

The partial GFADV genome was 4,441 nt in length with a low guanine–cytosine content of 37%. Similar to that of AMDV, the GFADV genome contained 2 major ORFs. The left ORF (LORF) contains the bulk of the sequences for the putative NS1, and the right ORF (RORF) codes for VP2 (Figure 1). Two small middle ORFs (67 and 75 aa long) with putative alternative start codons were detected in the 448-bp region between the ORFs. The theoretical proteins showed 55% and 59% aa identity with the 2 similarly located middle ORFs reported in AMDV. The partial 5′ untranslated region (UTR) was 109 nt and the partial 3′ UTR was 191 nt. Potential RNA splicing signals in AMDV were present on the GFADV genome (Technical Appendix Figure 1) (*4*). The predicted spliced transcripts encode hypothetical NS1, NS2, and NS3 of 635 aa, 115 aa, and 80 aa, respectively, and a capsid protein VP1 of 674 aa. The putative VP2 was predicted to arise from the intact transcript of RORF encoding a 630-aa protein.

Sequence analyses confirmed that GFADV was a divergent amdovirus with 76% nt identity with the genome of AMDV. Conserved protein domains typical of parvoviruses were identified in GFADV. In the LORF, the GKRN domain was found (Technical Appendix Figure 2), which may act as the nuclear transport signal of NS1 protein. In the RORF, 3 conserved domains (TPW, YNN, and PIW) of unknown biologic significance were detected (*6**,**14*) (Technical Appendix Figure 3). The phosholipase 2 motif in the N terminal VP1 region, generally conserved in parvoviruses, was not found in either GFADV not AMDV (Technical Appendix Figure 3), which suggests that this parvovirus genus uses a different mechanism to escape the endosome during infection (*15*).

**Figure 2:**
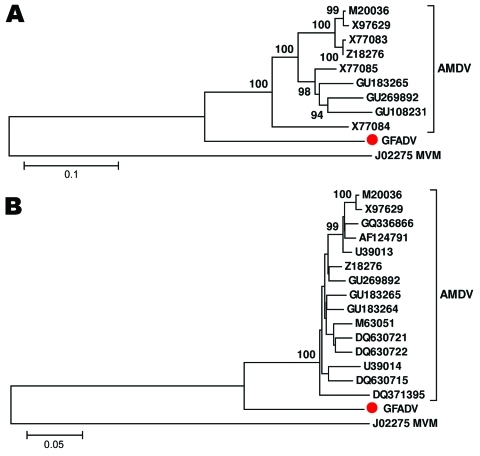
Phylogenetic analyses of gray fox amdovirus (GFADV) (red dots) and Aleutian mink disease virus (AMDV) based on the complete amino acid sequence of nonstructural protein 1 region (A) and viral protein 1 region (B). The neighbor-joining method was used with *p*-distance and 1,000 bootstrap replicates. Scale bars represent estimated phylogenetic divergence. GenBank accession numbers are shown on the tree. Minute virus of mice (MVM) was included as an outgroup.

Comparison of NS1 regions showed GFADV shared ≈74% nt and 67% aa similarities with AMDV strains, whereas different strains of AMDV shared >87% nt and 82% aa similarities. Alignments of the VP2 region showed GFADV shared ≈78% nt and 80% aa similarities with AMDV strains, whereas strains of AMDV had >92% nt and 91% aa similarities (Technical Appendix Figure 4, Technical Appendix Figure 5). To determine the relationship between GFADV and AMDV strains, phylogenetic analyses of the NS1 and VP2 proteins were performed, which showed that in both genome regions GFADV was more closely related to AMDV strains than to those of minute virus of mice or other parvoviruses analyzed (data not shown), but was distinct from the 3 AMDV groups (Figure 2). Pending review by the International Committee on Taxonomy of Viruses, GFADV thus appears to be the second reported parvovirus species in the genus *Amdovirus*.

GFADV sequences were also detected in the lung and heart tissues of the same animal by using a GFADV-specific nested PCR targeting a ≈400-bp segment of the VP2 gene, as well as in the heart tissue of another gray fox, which had the same signs, collected in Sonoma County in summer 2009. Further PCR screening of 19 tissue samples, including spleen, lung, liver, lymph node, and muscle from 9 other gray foxes with similar gait abnormalities and chronic muscle lesions, collected in 2008 (n = 2) and 2010 (n = 7) were negative by the same GFADV PCR.

## Conclusions

We report the identification and nearly complete genome sequence of an amdovirus found in the spleen, lung, and heart tissues of 2 gray foxes that exhibited an abnormal gait and muscle inflammation of unknown origin. On the basis of phylogenetic analyses, we propose this virus as the prototype member of a second species in the *Parvoviridae* genus *Amdovirus*. Putative NS and VP1/VP2 gene RNA splicing sites were detected in the GFADV genome, which suggests the expression of different NS and VP proteins.

Except for the ubiquitous anellovirus, GFADV was the only eukaryotic virus found in the spleen tissue of the diseased gray fox. The same virus was also identified in the heart tissue of a second gray fox collected the same summer but was not detected in tissues of 9 other gray foxes with a similar syndrome collected in different years. It is possible that GFADV is an incidental finding unrelated to these foxes’ symptoms. The lack of detectable GFADV DNA in all gray foxes with similar symptoms may also be because different tissues were compared in some animals or because tissue collection occurred at different stage of infection. Future testing of a possible link between GFADV and additional unexplained diseases of foxes and other carnivores will be facilitated by the availability of its genome sequence.

## Supplementary Material

Technical Appendix Nucleotide sequence around the predicted RNA splicing sites in the gray fox amdovirus genome.
