# Supplementary material for: Novel Amdovirus in Gray Foxes
Source: Emerg Infect Dis. 2011 Oct;17(10):1876–8. doi: 10.3201/eid1710.110233 (PMC3310670; doi:10.3201/eid1710.110233)
Supplement: Technical Appendix — Nucleotide sequence around the predicted RNA splicing sites in the gray fox amdovirus genome. [file 11-0233-Techapp_5p.pdf]

Article DOI: http://dx.doi.org/10.3201/eid1710.110233

Novel Amdovirus in Gray Foxes

Technical Appendix

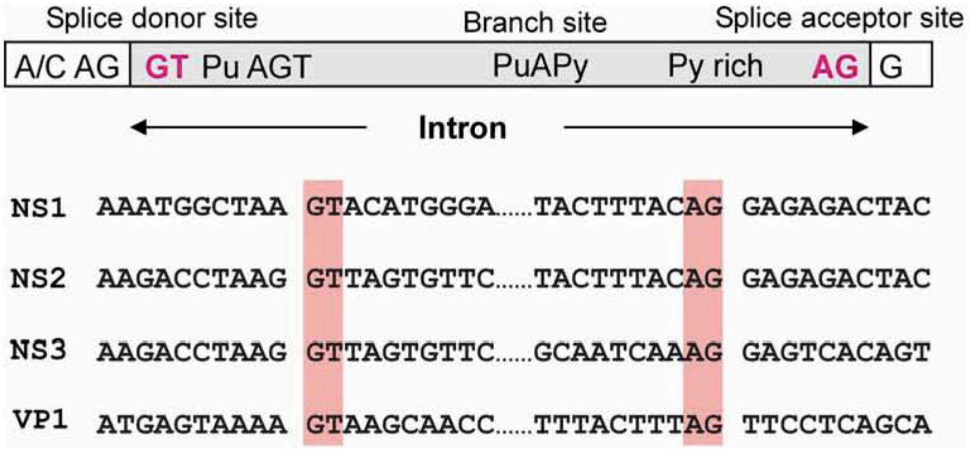

Technical Appendix Figure 1. Nucleotide sequence around the predicted RNA splicing sites in the gray fox amdovirus genome. Splice donor and receptor are shown in pink boxes. NS, nonstructural; VP, viral protein.

```
GFADV    1 MAQAQIAEQKILQALFEQLKKEIADGEGLAWLFQQKAYTDGDNKPTKETPPLRTTSEDLRLVFDSIEEN---------IK
AMDV     1 MAQAQIDEQRRLQDLYVQLKKEINDGEGVAWLFQQKTYTDKDNKPTKATPPLRTTSSDLRLAFDSIEEN---------LT
MVM      1 MISGSGSLNQGAKRKWAWFKVYKQLLKSVTYLFFHSVSRDAQKESNQLTMAGNAYSDEVLGATNWLKEKSNQEVFSFVFK
BPV      1 -MQPSRGLYSALHSFKHLLITIRQRTHAPDTVLSRDELNAWNWLQKIAQRVMRGDMDDIVDILKCRKAN-----------
AAV2     1 -----------MPGFYEIVIKVPSDLDEHLPGISDSFVNWVAEKEWELPPDSDMDLNLIEQAPLTVAEK-----------

GFADV   72 SNTDCLSNNEI---NFCKLTLGKTLVSLDKHIKSHRWNANNLQFIWQVEKGKTKHLHIHCLVG---YSDSTADKKDISKS
AMDV    72 ASNEHLTNNEI---NFCKLTLGKTLLLIDKHVKSHRWDSNKVNLIWQIEKGKTQQFHIHCCLG---YFDKNEDPKDVQKS
MVM     81 NENVQLNGKDIGWNSYKKELQEDELKSLQRGAETTWDQSEDMEWETTVDEMTKKQVFIFDSLVKKCLFEVLNTKNIFPGD
BPV     69 --------------------------------------------------------------------------------
AAV2    59 ---------------------------LQRDFLTEWRRVS-------------------------------------KAP

GFADV  146 LSWFIKKINKELATIWSNHHCTLQDIKRPEDR--QQNLNVWLQDGITKPYKYFNKQTRQEYNKQVNLREYTLIYLFDKNK
AMDV   146 LGWFMKRLNKDLAVIYSNHHCDIQDIKDPEDR--AKNLKVWIEDGPTKPYKYFNKQTKQDYNKPVHLRDYTFIYLFNKDK
MVM    161 VNWFVQHEWGKDQGWHCHVLIGGKDFSQAQGKWWRRQLNVYWSRWLVTACNVQLTPAERIKLREIAEDNEWVTLLTYKHK
BPV     69 -GTLVAQAINGTEFITRYMLPKNR------------------------------KVADTVLTRHTTPEQ--------SYD
AAV2    75 EALFFVQFEKGESYFHMHVLVETTGVKSMVLG------------------RFLSQIREKLIQRIYRGIEPTLPNWFAVTK

GFADV  224 ITEKGMDGYFAAGNGGFIDNMTEKERKIMRKMYLDEQCSDMADDSQDWDDSQEPPKTEPPKVTTETISNVTYVDSAVVQP
AMDV   224 INTDSMDGYFAAGNGGIVDNLTNKERKTLRKMYLDEQSSDIMDANIDWEDGQ-----DAPKVTDQT-------DSATTKT
MVM    241 QTKKDYTKCVLFGN--MIAYYFLTKKKISTSPPRD--GGYFLSSDSGWKTNF---------LKEGE-------RHLVSKL
BPV    110 STWGKTYGFAVCNGETVSEFTRKDLWKVLYNIYTAHPAENMLNSN--------------------------------PSV
AAV2   137 TRNGAGGGNKVVDECYIPNYLLPKTQPELQWAWTN--MEQYLSACLNLTERK-----------------------RLVAQ

GFADV  304 GTSTIWASCSTKATKPKTITETAKQPSKKLTTAKSTLDSLFDIGCFTPEDMILKLSDTYLELSLEANGTNKILTLLHMNQ
AMDV   292 GTSLIWKSCATKVTSKKEVANPVQQPSKKLYSAQSTLDALFNVGCFTPEDMIIKQSDKYLELSLEPNGPQKINTLLHMNQ
MVM    301 YTDDMRPETVETTVTTAQETKRGRIQTKKEVSIKTTLKELVHKRVTSPEDWMMMQPDSYIEMMAQPGGENLLKNTLEICT
BPV    158 WGDLPRVSANRIDADDAEARSRPIKLSRKQKIMAEVIQRATDGLLLTYNDLVVHLSDLMLMLEGMPGGSKTAEQLLTMIH
AAV2   192 HLTHVSQTQEQNKENQNPNSDAPVIRSKTSARYMELVGWLVDKGITSEKQWIQEDQASYISFNAASNSRSQIKAALDNAG

GFADV  384 VKTASILNAFECIMKFNDDE------------DEKPLINIINQMGLNEKVLKNIIATVLTKQSGKRGCIWFYGPGGTGKT
AMDV   372 VKTSTMITAFDCIIKFNEEE------------DDKPLLATIKDMGLNEQYLKKVLCTILTKQGGKRGCIWFYGPGGTGKT
MVM    381 LTLARTKTAFDLILEKAETSKLT-----NFSLPDTRTCRIFAFHGWNYVKVCHAICCVLNRQGGKRNTVLFHGPASTGKS
BPV    238 IKLCAKYNAYEFMLMKTPATQNMNPGAPHYDCQGNLVFKLLNLQGYNPWQVGHWLVMMLSKKTGKRNSTLFYGPASTGKT
AAV2   272 KIMSLTKTAPDYLVGQQPVED----------ISSNRIYKILELNGYDPQYAASVFLGWATKKFGKRNTIWLFGPATTGKT

GFADV  452 LLANLICTAVKNFGMVTTSNQNFPWTDCGNRNMIWLEECGNLGNFIEDFKAITGGGDIKVDTKNKQPQAIKG-VTVITSN
AMDV   440 LLASLICKATVNYGMVTTSNPNFPWTDCGNRNIIWAEECGNFGNWVEDFKAITGGGDVKVDTKNKQPQSIKG-CVIVTSN
MVM    456 IIAQAIAQAVGNVGCYNAANVNFPFNDCTNKNLIWVEEAGNFGQQVNQFKAICSGQTIRIDQKGKGSKQIEPTPVIMTTN
BPV    318 NLAKAICHAVGLYGCVNHNNKQFPFNDAPNKMILWWEECIMTTDYVEAAKCVLGGTHVRVDVKHKDSRELPQIPVLLSSN
AAV2   342 NIAEAIAHTVPFYGCVNWTNENFPFNDCVDKMVIWWEEGKMTAKVVESAKAILGGSKVRVDQKCKSSAQIDPTPVIVTSN

GFADV  531 KDITKVTIGAVETNVHSEPLKQRIVKIRCVKTINPTTKITP---------------------------------------
AMDV   519 TNITKVTVGCVETNAHAEPLKQRMIKIRCMKTINPKTKITP---------------------------------------
MVM    536 ENITVVRIGCEERPEHTQPIRDRMLNIHLTHTLPGDFGLVDK-----------------NEWPMICAWLVKNGYQSTMAS
BPV    398 HDVYTVVGGNATFGVHAAPLKERITQMNFMKQLPNTFGEITPGMISNWLSHCAHIHQEHLSLEGFAIKWDVQSVGNSFPL
AAV2   422 TNMCAVIDGNSTTFEHQQPLQDRMFKFELTRRLDHDFGKVTK-----------------QEVKDFFRWAKDHVVEVEHEF

GFADV  572 -----GMLKKW--------------------LSTWEG----KPIQLNHEMENLYRSK-----------------------
AMDV   560 -----GMLKRW--------------------LNTWDR----QPIQLSHEMPELYLGKCRW--------------------
MVM    599 YCAKWGKVPDW--------------------SENWAEPKVPTPINLLGSARSPFTTPKSTPLSQN---------------
BPV    478 QTLCPGHSQNWTFSENGVCWHCGGFIQPTPESDTDSDGDPDPDGAVAGDSDTSANSESTVSFSSNDSGLGSVTSSAPSVP
AAV2   485 YVKKGGAKKRP--------------------APSDADISEPKRVRESVAQPSTSDAEASINYADR---------------

GFADV      --------------------------------------------------------------------------------
AMDV       --------------------------------------------------------------------------------
MVM    644 --------------------------YALTPLASDLEDLALEPWS------------TPNTPVAGTAETQNTGEAGSKAC
BPV    558 DRAEEIEEIPSECLEWMREEVDRLSAHDINSLAHQATGFILDPIPEEPEEGERDLAREDAEPEASTSHTPATKRARVEEG
AAV2   530 --------------------------YQNKCSRHVGMNLMLFPCR------------QCERMNQNSNICFTHGQKDCLEC

GFADV      --------------------------------------------------------------------------------
AMDV       --------------------------------------------------------------------------------
MVM    686 QDGQLSPTWSEIEEDLR--------------ACFGAEPLKKDFSEPLNLD------------------------------
BPV    638 EPWDGTQPITEGDWIDFESRQKRRRLEREEKGGEDEDMEVQESDPSAWGEKLGIVEKPGEEPIVLYCFETLPESDEEGDS
AAV2   572 FPVSESQPVSVVKKAYQKLCYIHHIMGKVPDACTACDLVNVDLDDCIFEQ------------------------------

GFADV      ---------
AMDV       ---------
MVM        ---------
BPV    718 DKENKTHTV
AAV2       ---------
```

Technical Appendix Figure 2. Conservative domain in the left open reading frame of gray fox amdovirus and representative parvoviruses. GKRN region is marked in red.

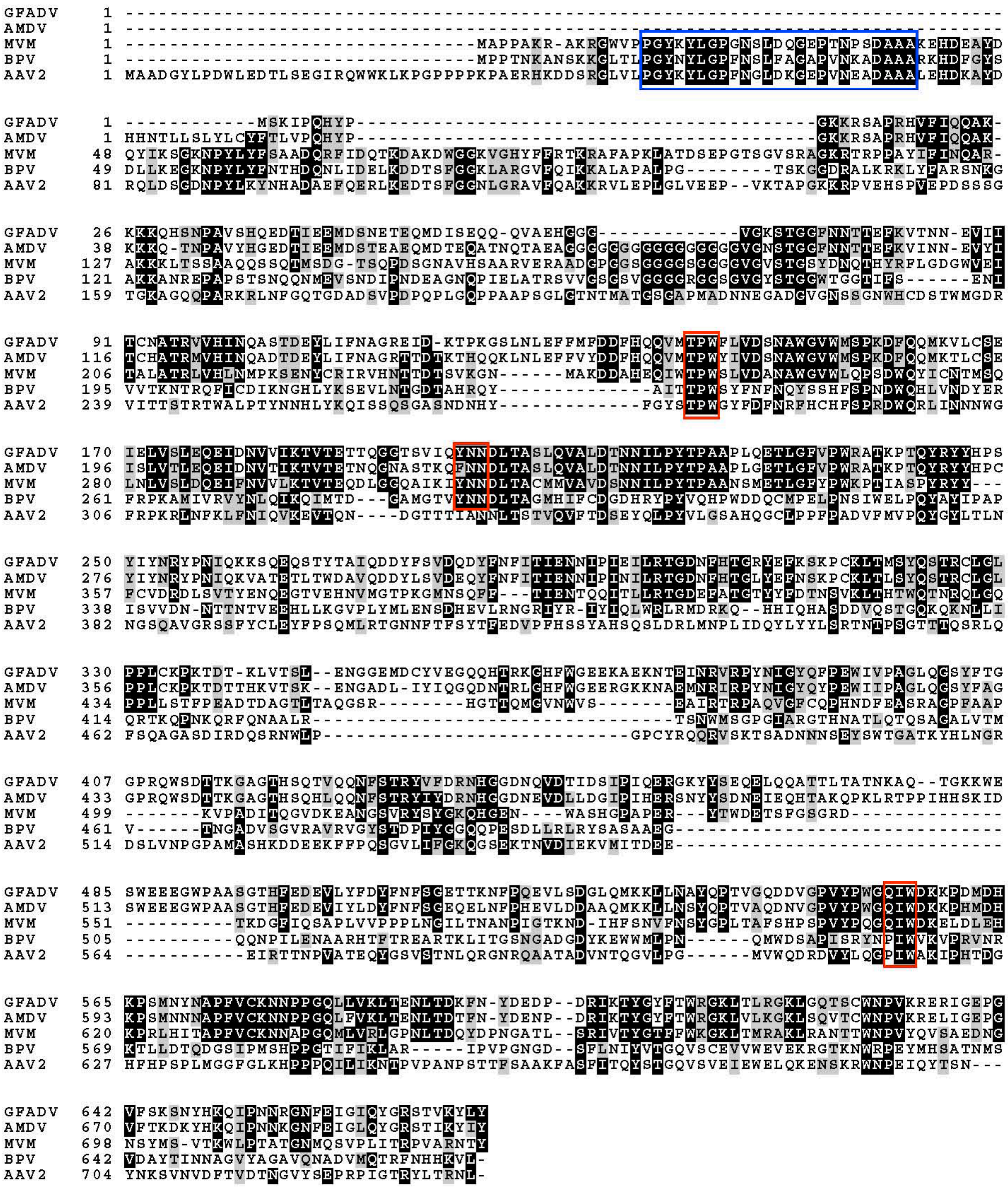

Technical Appendix Figure 3. Conservative domains in the right open reading frame of gray fox amdovirus and representative parvoviruses. PGY region (putative phopholipases A2 domain) is marked in blue and TPW, YNN, and PIW region are marked in red.

```
GFADV     1  MAQAQIAEQKILQALFEQLKKEIADGEGLAWLFQQKAYTDGDNKPTKETPPLRTTSEDLRLVFDSIEENIKSNTDCLSNN
M20036    1  MAQAQIDEQRRLQDLYVQLKKEINDGEGVAWLFQQKTYTDKDNKPTKATPPLRTTSSDLRLAFDSIEENLTASNEHLTNN
X77084    1  MAQAQLNEQRRLQELFEKFKTEVADGEGLAWLFQQKTYTDKDNKPTKATPPLRTTSTDLRLAFDSIEETLKTSNQCLTNN
X77085    1  MAQAQLDEQKRLQDLYTQLKKEVADGEGLAWLFQQKTYTDKDNKPTKATPPLRTTSSDLRLAFDSIEETLIASNEWLTKD

GFADV    81  EINFCKLTLGKTLVSLDKHIKSHRWNANNLQFIWQVEKGKTKHLHIHCLVGYSDSTADKKDISKSLSWFIKKINKELATI
M20036   81  EINFCKLTLGKTLLLIDKHVKSHRWDSNKVNLIWQIEKGKTQQFHIHCCLGYFDKNEDPKDVQKSLGWFMKRLNKDLAVI
X77084   81  DINFCKLTLGKTLVLLDKHVRSHRWDANKVNFIWQVEKGKTQQFHIHCCLGYFDKDEDSKDVQKSLGWFIKKLNKDLAVI
X77085   81  EINFCKLTLGKTLVLVDKHVKSHRWDADKINFIWQIEKGKTQHFHIHCCLGYFDKNEDPKDVQKSLGWLIKKLNRDLAAI

GFADV   161  WSNHHCTLQDIKRPEDRQQNLNVWLQDGITKPYKYFNKQTRQEYNKQVNLREYTLIYLFDKNKITEKGMDGYFAAGNGGF
M20036  161  YSNHHCDIQDIKDPEDRAKNLKVWIEDGPTKPYKYFNKQTKQDYNKPVHLRDYTFIYLFNKDKINTDSMDGYFAAGNGGI
X77084  161  WSNHHCDIQGIQDSDGRADNLKIWIEDGPTKPYKYFNKQTKQDYNKPTNLRDYTLIYLFNKDKITQEGMDGYYASGNGGI
X77085  161  FSNHHCDQQDIKDPEAKANNLKVWIEDGPTKPYKYYNKQTKQDYNKPVTLSDYVIIYLFNKDKIHKEGMDGYYAAGNGGL

GFADV   241  IDNMTEKERKIMRKMYLDEQCSDMADDSQDWDDSQEPPKTEPPKVTTETISNVTYVDSAVVQPGTSTIWASCSTKATKPK
M20036  241  VDNLTNKERKTLRKMYLDEQSSDIMDANIDWEDGQD-----APKVTDQT-------DSATTKTGTSLIWKSCATKVTSKK
X77084  241  IDNLTNKERKALRKMYLDEQSQGILDEDIDWEDSQS-----APKVTDQT-------DSATSKTGTSLVWKSCATKVTSKK
X77085  241  IDNLTNKERKALRKMYLDEQSSDIMDADIDWEDGQD-----APKVTDQT-------DSATSKTGTSLIWKSCATKVTSKK

GFADV   321  TITETAKQPSKKLTTAKSTLDSLFDIGCFTPEDMILKLSDTYLELSLEANGTNKILTLLHMNQVKTASILNAFECIMKFN
M20036  309  EVANPVQQPSKKLYSAQSTLDALFNVGCFTPEDMIIKQSDKYLELSLEPNGPQKINTLLHMNQVKTSTMITAFDCIIKFN
X77084  309  EVAIPVKQPSKKMTSAQNTLDDLYMFGCFTPEDMILKMSDRYLEMSLEPNGAQKINTLLHMNQVRTSSNLTAFECIIKFN
X77085  309  EVANPVQQPSKKLYSAQNTLDALFNVGCFTPEDMIIKQSDKYLELSLEPNGPQKINTLLHMNQVKTSTMMTAFDCIIKFN

GFADV   401  DDEDEKPLINIINQMGLNEKVLKNIIATVLTKQSGKRGCIWFYGPGGTGKTLLANLICTAVKNFGMVTTSNQNFPWTDCG
M20036  389  EEEDDKPLLATIKDMGLNEQYLKKVLCTILTKQGGKRGCIWFYGPGGTGKTLLASLICKATVNYGMVTTSNPNFPWTDCG
X77084  389  EEEDEKPLLDTIKDMGLNEQHLKKVLCTILTKQSGKRGCVWFYGPGGTGKTLLASLICKAVVNYGMVTTSNPNFPWTDCG
X77085  389  EEEDDKPLLATIKDMGLNEQYLKKVLCTILTKQGGKRGCIWFYGPGGTGKTLLASLICKATVNYGMVTTSNPNFPWTDCG

GFADV   481  NRNMIWLEECGNLGNFIEDFKAITGGGDIKVDTKNKQPQAIKGVTVITSNKDITKVTIGAVETNVHSEPLKQRIVKIRCV
M20036  469  NRNIIWAEECGNFGNWVEDFKAITGGGDVKVDTKNKQPQSIKGCVIVTSNTNITKVTVGCVETNAHAEPLKQRMIKIRCM
X77084  469  NRNIIWAEECGNIGNYVEDFKAITGGGDVKVDTKNKQPQSIKGSVIVTSNTNITKVTVGCVETNVHAEPLKQRMVKIRCM
X77085  469  NRNIIWAEECGNLGNWVEDFKAITGGGDVKVDTKNKQPQSIKGCVIVTSNTNITKVTVGCVETNAHAEPLKQRMIKIRCM

GFADV   561  KTINPTTKITPGMLKKWLR------------------ETTGANYPVTSAEKNTGFLQPSTAESAGGANTTKSDTQRKVVD
M20036  549  KTINPKTKITPGMLKRWLNTWDRQPIQLSHEMPELYLETTGPNSSATTATKNTGNSQPTTAKSAESVNTENCDTPKRSAS
X77084  549  KPVNPKTKVTPGMLKTWISTWDRIPIKLSHEMPELYLETSGPNSSATTATKSTGNLQPTTAETAESVNTANCDTPKRGAS
X77085  549  KTINPKTKITPGMLKRWLNTWDRQPIQLSHEMPELYLETTGPNSSATSATKTTGNSQPTTAKTAESVSTADCDTPKRGAS

GFADV   623  SAPLKAAKRPRHE
M20036  629  SVPAKQHKRPRHE
X77084  629  SVPPKQHKRPRHE
X77085  629  SVPPKQHKRPRHE
```

Technical Appendix Figure 4. Nonstructural protein 1 alignment of gray fox amdovirus and representative amdoviruses. Black highlights 70%–100% similarity, gray highlights 50–70% similarity.

```
GFADV       1 MDSNETEQMDISEQQ-QVAEHGG-------------GVGKSTGGFNNTTEFKVTNNEVIITCNATRVVHINQASTDEYLI
M20036      1 MDSTEAEQMDTEQATNQTAEAGGGGGGGGGGGGGGGGVGNSTGGFNNTTEFKVINNEVYITCHATRMVHINQADTDEYLI
GU183265    1 MDSTEPEQMDTEQATNQTAEAGGGGGG-GGGGGGGGGVGNSTGGFNNTTEFKVINNEVYITCHATRMVHINQADTDEYLI
DQ371395    1 MDSTEPEQMDTEQATNQTAEAGGGGGGSGGGGGGGGGVGNSTGGFNNTTEFKVINNEVYITCHATRMVHINQADTDEYLI

GFADV      67 FNAGREID-KTPKGSLNLEFFMFDDFHQQVMTPWFLVDSNAWGVWMSPKDFQQMKVLCSEIELVSLEQEIDNVVIKTVTE
M20036     81 FNAGRTTDTKTHQQKLNLEFFVYDDFHQQVMTPWYIVDSNAWGVWMSPKDFQQMKTLCSEISLVTLEQEIDNVTIKTVTE
GU183265   80 FNAGRTTDTKTAQKKLDLEFFVYDDFHQQVMTPWFLVDSNAWGVWMSPKDFQQMKTLCSEISLVTLEQEIDNVTIKTVTE
DQ371395   81 FNAGRTTDTKTAQKKLNLEFFVYDDFHQQVMTPWFLVDSNAWGVWMSPKDFQQMKTLCSDISLISLEQEIDNVTIKTVTE

GFADV     146 TTQGGTSVIQYNNDLTASLQVALDTNNILPYTPAAPLQETLGFVPWRATKPTQYRYYHPSYIYNRYPNIQKKSQEQSTYT
M20036    161 TNQGNASTKQFNNDLTASLQVALDTNNILPYTPAAPLGETLGFVPWRATKPTQYRYYHPCYIYNRYPNIQKVATETLTWD
GU183265  160 TNQGNASTKQFNNDLTASLQVALDTNNILPYTPAAPLGETLGFIPWRATKPTQYRYYHPCYIYNRYPNIQKTGQQQLEWT
DQ371395  161 TNQGNASTKQFNNDLTASLQVALDTNNILPYTPAAPLGETLGFVPWRATKPTQYRYYHPCYIYNRYPNIQKTG-QELEWT

GFADV     226 AIQDDYFSVDQDYFNFITIENNIPIEILRTGDNFHTGRYEFKSKPCKLTMSYQSTRCLGLPPLCKPKTD-TKLVTSLENG
M20036    241 AVQDDYLSVDEQYFNFITIENNIPINILRTGDNFHTGLYEFNSKPCKLTLSYQSTRCLGLPPLCKPKTDTTHKVTSKENG
GU183265  240 GTQDDYLSVDEQYFNFITVENNIPINILRTGDNFHTGLYEFKSKPCKLTLSYQSTRCLGLPPLCKPKTDVTHKVTSLQNG
DQ371395  240 GTQDDYLSVDEQYFNFITIENNIPINILRTGDNFHTGLYEFKSKPCKLTFSYQSTRCLGLPPLCKPKTDKTHKATSKENG

GFADV     305 GEMDCYVEGQQHTRKGHFWGEEKAEKNTEINRVRPYNIGYQFPEWIVPAGLQGSYFTGGPRQWSDTTKGAGTHSQTVQQN
M20036    321 ADL-IYIQGQDNTRLGHFWGEERGKKNAEMNRIRPYNIGYQYPEWIIPAGLQGSYFAGGPRQWSDTTKGAGTHSQHLQQN
GU183265  320 ADL-IYIEGQDNTRLGHFWGEERGKKNAEMNRVRPYNIGFQYPEWIIPAGLQGSYFAGGPRQWSDTTKGAGTHSQQLQQN
DQ371395  320 ADL-IDVQRQRNTRLGHFWGEQRGKKNAEMNRIRPYNIGNQYPEWIIPAGLKGSYFAGGQRQWSDTTKGAGTHSQNLKQN

GFADV     385 FSTRYVFDRNHGGDNQVDTIDSIPIQERGKYYSEQELQQATTLTATNKAQ--TGKKWESWEEEGWPAASGTHFEDEVLYF
M20036    400 FSTRYIYDRNHGGDNEVDLLDGIPIHERSNYYSDNEIEQHTAKQPKLRTPPIHHSKIDSWEEEGWPAASGTHFEDEVIYL
GU183265  399 FSTRYIYDRNHGGDNEVDLLDGIPIHERSNYYSDHEIEQHTAKQPKLRVPPIHHSKIDSWEEEGWPAASGTHFEDEVIYL
DQ371395  399 FSTRYIYDRNNGGDKEVNLLDGIPIHERSKYYSDNEIEQHTAKQPKLRTPPIHHSKIDSWEEEGWPAASGTHFEDEVIYL

GFADV     463 DYFNFSGETTKNFPQEVLSDGLQMKKLLNAYQPTVGQDDVGPVYPWGQIWDKKPDMDHKPSMNYNAPFVCKNNPPGQLLV
M20036    480 DYFNFSGEQELNFPHEVLDDAAQMKKLLNSYQPTVAQDNVGPVYPWGQIWDKKPHMDHKPSMNNNAPFVCKNNPPGQLFV
GU183265  479 DYFNFGGEQTLEFPHEVLDDAAQMKKLLNSYQPTVAQDNVGPVYPWGQIWDKKPDMDHKPSMNNNAPFVCKNNPPGQLFV
DQ371395  479 DYFNLSGETELEFPHEVLDDAAQMKKLLNSYQPTVALDNVGPVYPWGQIWDKKPDMDHKPSMNNSASFVCKNNPPGQLFV

GFADV     543 KLTENLTDKFNYDEDPDRIKTYGYFTWRGKLTLRGKLGQTSCWNPVKRERIGEPGVFSKSNYHKQIPNNRGNFEIGIQYG
M20036    560 KLTENLTDTFNYDENPDRIKTYGYFTWRGKLVLKGKLSQVTCWNPVKRELIGEPGVFTKDKYHKQIPNNKGNFEIGLQYG
GU183265  559 KLTENLTDTFNYDENPDRIKTYGYFTWRGKLVLKGKLSQVTCWNPVKRELIGEPGVFTKDNYHKQIPNNKGNFEIGLQYG
DQ371395  559 KLTENLTDTFNYDENPDRIKTYGYFTWRGKLVIKGKLSQVTCWSPVKRELIGEPGVFTKDNYHKQIPNNKGNYEIGLQYG

GFADV     623 RSTVKYLY
M20036    640 RSTIKYIY
GU183265  639 RSTIKYIY
DQ371395  639 RSTIKYIY
```

Technical Appendix Figure 5. Viral protein 2 alignment of gray fox amdovirus and representative amdoviruses. Black highlights 70%–100% similarity, gray highlights 50–70% similarity.
